# MASQOT: a method for cDNA microarray spot quality control

**DOI:** 10.1186/1471-2105-6-250

**Published:** 2005-10-13

**Authors:** Max Bylesjö, Daniel Eriksson, Andreas Sjödin, Michael Sjöström, Stefan Jansson, Henrik Antti, Johan Trygg

**Affiliations:** 1 Research group for Chemometrics, Department of Chemistry, Umeå University, SE-901 87 Umeå, Sweden; 2 Umeå Plant Science Centre, Department of Forest Genetics and Plant Physiology, Swedish University of Agricultural Sciences, SE-901 83 Umeå, Sweden; 3 Umeå Plant Science Centre, Department of Plant Physiology, Umeå University, SE-901 87 Umeå, Sweden

## Abstract

**Background:**

cDNA microarray technology has emerged as a major player in the parallel detection of biomolecules, but still suffers from fundamental technical problems. Identifying and removing unreliable data is crucial to prevent the risk of receiving illusive analysis results. Visual assessment of spot quality is still a common procedure, despite the time-consuming work of manually inspecting spots in the range of hundreds of thousands or more.

**Results:**

A novel methodology for cDNA microarray spot quality control is outlined. Multivariate discriminant analysis was used to assess spot quality based on existing and novel descriptors. The presented methodology displays high reproducibility and was found superior in identifying unreliable data compared to other evaluated methodologies.

**Conclusion:**

The proposed methodology for cDNA microarray spot quality control generates non-discrete values of spot quality which can be utilized as weights in subsequent analysis procedures as well as to discard spots of undesired quality using the suggested threshold values. The MASQOT approach provides a consistent assessment of spot quality and can be considered an alternative to the labor-intensive manual quality assessment process.

## Background

At present, the DNA microarray technology allows simultaneous monitoring of the expression levels of thousands of genes. The technique produces large and complex datasets that are relatively easy to generate but non-trivial to analyze and extract information from. So far, much of the data mining efforts have been focused on the statistical analysis (see, for instance [1-4]) and less on acquiring high quality data from the image analysis. Image analysis is the process of extracting information from the scanned microarray images, which is an important step due to the sequential nature of the microarray analysis [5]. Consequently, problems in the initial steps have large impact on the interpretation of the final results from the experiment.

A number of technical issues during microarray preparation potentially affect the spot quality.

• *Low signal intensity *is perhaps the most generally acknowledged property that affects spot quality due to the subsequent problems in distinguishing signal from noise for spots with weak signals. Weak signal intensities should result from physiologically low expression levels but might also be related to surface properties of the slide, signal bleaching, scanner problems or incomplete or irregular hybridization.

• *Intensity distribution issues *appear as regions of pixels containing signals that clearly deviate from the average signal, typically as distinct sub-areas within the foreground region. As the signal of any given spot on a microarray slide is expected to be uniform over the entire spot foreground area, intensity distribution issues are usually a consequence of non-specific binding or irregular distribution of the printed DNA on the slide.

• *Morphological issues *refer to unexpected shape-related variations of the spot foreground region. This includes very small or very large spot sizes, low spot circularities or spot mixing. Size aberrations might be a consequence of precipitates or impurities in the printing solution or needle clogging during printing. Spots are expected to be roughly circular in shape, but manufacturing issues might result in deviation from the circularity norm. Furthermore, imperfections on the slide or washing problems might cause the dye from several spots to mix, referred to as *bleeding*, making the separation of these signals difficult or even impossible.

• *Background issues *appear as intensity fluctuations in the local background region immediately surrounding the foreground region. An increase in local background intensity or variance compared to the global slide background typically result from dye contaminants due to non-specific binding or incomplete washing.

Microarray spot quality control is essentially the identification and removal of spots with properties that cause the subsequent interpretation of the signal from these spots to be unreliable or misleading. Analogously, it is the recognition of characteristics that enable dependable interpretations and conclusions from the generated data. The field of microarray spot quality control has been largely neglected in the past but has recently become an area of interest. Existing documented semi-automatic methodologies include Bayesian networks [6] as well as linear combinations of quality parameters allegedly related to the quality of the spot [7-10]. Interestingly, manual evaluation is still a common quality assessment procedure, despite the labor-intensive nature of visually inspecting spots in the order of hundreds of thousands. Increasing availability and usage of microarrays in the field of transcriptomics has caused experiments to involve an escalating number of slides, which in turn has highlighted the primary bottleneck of manual quality assessment.

Partial least squares (PLS) is a generalized regression method which aims to maximize the covariance between the **X **(descriptor) and **Y **(response) matrices. PLS can handle large data sets of multi-collinear and noisy data with moderate amounts of missing data in both **X **and **Y**. PLS-DA can be seen as a special case of PLS where the response matrix **Y **is categorical (numerically represented as 0 or 1) and determines class belonging of observations. PLS-DA has been widely applied in microarray analysis (see, for instance [11,12]) as well as other areas of life science (see, for instance [13,14]). For a more detailed description of the properties of PLS, please consult [15-17] and references therein.

**Figure 1:**
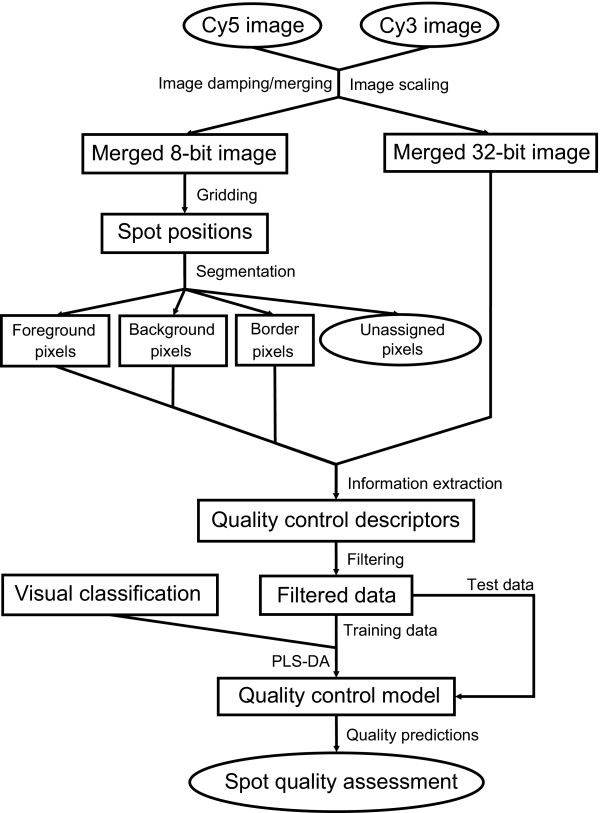
**Flowchart of the classification procedure**. The classification process involves an 8-bit image, optimized for segmentation, as well as a 32-bit image, used for information extraction. During the training phase, visual classification results are required while this is not necessary for external data.

Here, we propose the microarray spot quality control (MASQOT) methodology for assessment of cDNA microarray spot quality, outlined in figure 1. A set of existing and novel spot descriptors were identified that aimed to characterize spot quality in terms of physical attributes of the spot. Prior to the extraction of descriptors, manual assessment of the spot quality was performed independently by three experienced microarray users on roughly eighty thousand spots in order to provide a sufficiently large data set of known quality.

Spot descriptors were subsequently subjected to multivariate discriminant analysis by means of PLS-DA with the aim to categorize spots of low quality and spots of high quality by treating these spots as separate classes. The utilized descriptors aimed to describe foreground and background irregularity measures, spot morphology and foreground density attributes that were potentially useful for discriminating between the reliable (not bad) and unreliable (bad) spots. For instance, the *circularity *measure is a descriptor ranging from 0% to 100%. If the circularity descriptor approaches its minimum value, the predicted class belonging should typically be higher (closer to 1) for the bad class compared to the not bad class. The *coefficient of variation *for the foreground and background regions is an example of an employed descriptor that illustrates reverse characteristics; higher values should typically provide greater class conformity with the bad class compared to the not bad class. However, all employed descriptors together contribute to the regression model and consequently to the final class determination at varying degrees depending on the properties of the spot.

The MASQOT approach aims to provide a consistent assessment of spot quality, applicable to various types of microarray data, thus avoiding the labor-intensive manual quality assessment process. The methodology generates continuous values of spot quality which can be utilized to discard spots of undesired quality or used as weights in subsequent analysis procedures.

## Results

Five cDNA microarray slides using the *Populus *second generation microarray slide layout (POP2) where the samples originate from a previous investigation of leaf development (Sjödin *et al*, in preparation) were used for classification training. Five additional POP2 slides, not included in the training set, were employed for external validation. Segmentation of raw images was performed using an implementation of the Seeded Region Growing (SRG) algorithm [18]. The properties of each spot were subsequently characterized using a large set of descriptors allegedly linked to spot quality. These properties include foreground and background variability measures, spot morphology and foreground intensity distribution measures. Please consult Additional file 1 for a complete list of all the utilized descriptors.

Following segmentation, spots were inspected by three experienced microarray users and independently assigned to the two quality categories {bad, not bad}. Spots in the bad category consisted of all the spots that were classified as bad by at least one of the experienced users while the remaining spots were categorized as not bad. For classification and evaluation purposes, the spots in the bad category were subsequently partitioned into different sub-classes based on visual properties as described in table 1. This can be seen as characterizing each spot as exhibiting

**Table 1:** The different sub-classes of bad spots.

**Class**	**Description**
not bad	No issue. Contains all spots with no apparent problems according to the classification by the three experienced users.
HIFI	High-Intensity Foreground Issue. Typically intensity distribution issues, such a dye debris in the foreground region or donut-shaped spots, with very distinct characteristics.
LIFI	Low-Intensity Foreground Issue. Weak intensity distribution issues in the foreground region or morphological issues.
HIBI	High-Intensity Background Issue. Typically intensity distribution issues, such a dye debris in the background region, with very distinct characteristics.
LIBI	Low-Intensity Background Issue. Weak intensity distribution issues or faint increases in noise level in the background region.
HIFI/HIBI	A combination of HIFI and HIBI.
HIFI/LIBI	A combination of HIFI and LIBI.
LIFI/HIBI	A combination of LIFI and HIBI.
LIFI/LIBI	A combination of LIFI and LIBI.
HIFI/LIFI	A combination of HIFI and LIFI.
HIFI/LIFI/HIBI	A combination of HIFI and LIFI and HIBI.

1. No issues (not bad); or

2. Foreground issues (FI); or

3. Background issues (BI); or

4. Any combination of 2 and 3

To avoid confounding of properties, only spots displaying pure issues (entries 1–3) were used in the classification training, although all spots were used in the model evaluation. The three-class problem was subjected to multivariate analysis by means of PLS [16] regression coupled with discriminant analysis (PLS-DA) with the aim to discriminate not bad spots from FI spots and BI spots. The result from the PLS-DA regression model is a predicted class conformity (CC) value for each of the classes: not bad (CC_nb_), foreground issues (CC_FI_) and background issues (CC_BI_) with the added restriction that CC_nb _= 1 - (CC_FI _+ CC_BI_). Due to this restriction, only the conformity value of the not bad spots (CC_nb_) will be interpreted in the upcoming sections. A CC_nb _value approaching 1 denotes high compliance with the not bad class, which can be interpreted as a quality measure of the spot. Spots visually categorized as bad should thus exhibit a value of CC_nb _close to 0 whereas spots categorized as not bad should exhibit a value of CC_nb _close to 1.

**Figure 2:**
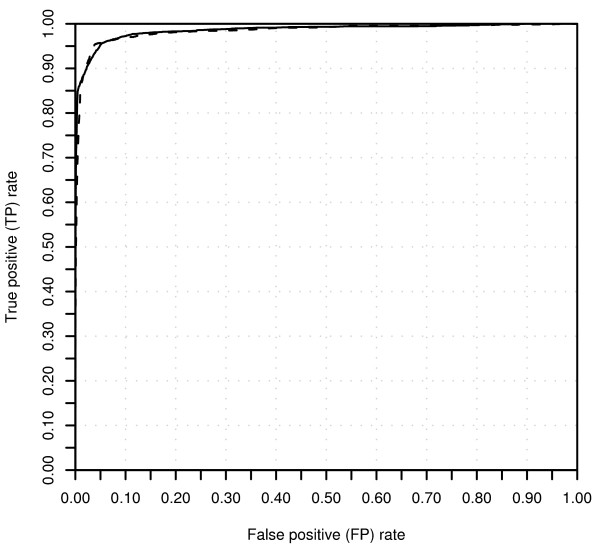
**Receiver Operating Characteristics (ROC) plot**. The relation between true positives (bad spots classified as bad) and false positives (not bad spots classified as bad) for the training and test data. The solid line denotes training data whereas the dashed line denotes test data.

**Figure 3:**
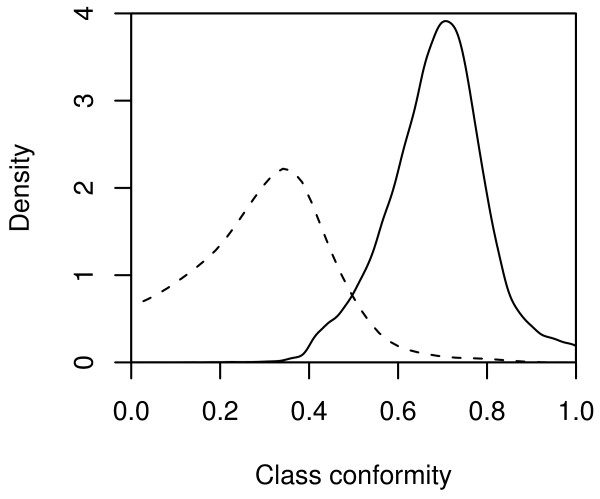
**Density plot of the predicted class conformity of the not bad class**. A class conformity value of 1 signifies perfect class conformity while a value of 0 signifies no class conformity. The dashed line illustrates the density for the prediction of the bad spots in the POP2 training set whereas the solid line illustrates the density of the prediction of the not bad spots in the POP2 training set.

Receiver Operating Characteristics (ROC) plot of the classifications of the POP2 training set and POP2 test set, respectively, is available in figure 2. A density plot of the CC_nb _value for the bad and not bad spots in the POP2 training set is shown in figure 3, showing a partial overlap between the discrimination of the two classes. Due to this overlap, discrimination accuracy was dependent on a threshold value *t *denoting the separation point between the two classes.

**Figure 4:**
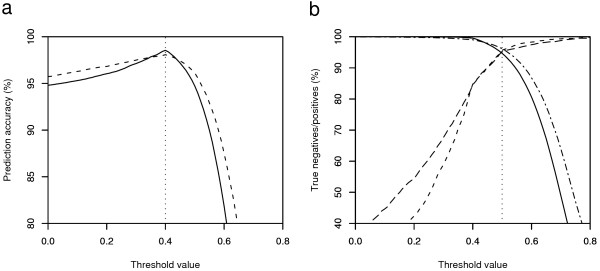
**Relationship between classification accuracy and threshold value for the POP2 data. **The threshold value *t *defines the boundary between bad and not bad spots for the POP2 training set (38 627 spots) and the POP2 test set (39 421 spots). Spots with a predicted class conformity value for the not bad class (CC_nb_) below the threshold value *t *are classified as bad while the remaining spots are classified as not bad. **a) **Overall classification accuracy *vs*. threshold value calculated as the fraction of correctly classified spots in the data set for a given threshold value. The solid line represents the POP2 training set whereas the dashed line represents the POP2 test set. The dotted vertical line at threshold value *t *= 0.4 illustrates an approximate maximum. **b) **Classification accuracy of the bad and not bad spots *vs*. threshold value. For the POP2 training set, the solid line represents the classification accuracy of the not bad spots and the dashed line represents the classification accuracy of the bad spots. For the POP2 test set, the dot-dashed line represents the classification accuracy of the not bad spots and the long-dashed line represents the classification accuracy of the bad spots. The dotted vertical line at threshold value *t *= 0.5 denotes the intersection point.

Spots with a predicted CC_nb _value below *t *were classified as bad whereas the remaining spots were classified as not bad. The threshold value can be set more or less stringently depending on the quality filtering requirements. This is illustrated in figures 4a–b, depicting different views of interpreting the classification accuracy of the POP2 training set and the POP2 test set. The threshold values were set either to maximize the overall classification accuracy or to maximize the class-wise classification accuracy. The overall classification accuracies for the POP2 training set (38 627 spots) and POP2 test set (39 421 spots) were calculated using equation 1 for all *t *in the interval (0,1) using CC_nb _values for the {bad, not bad} spots. The classification accuracy peak at a level of approximately 98% where *t *= 0.4 (see figure 4a). Predicted class-wise accuracies were calculated using equation 1 employing CC_nb _values for the bad spots and CC_nb _values for the not bad spots separately. The predicted class-wise accuracies intersect at a level of 95% (see figure 4b) for *t *= 0.5. Exact classification accuracies per sub-class, based on the intersection threshold value *t *= 0.5 as illustrated in figure 4b, are available in table 2.

**Table 2:** Classification accuracy of the POP2 training data. The classification accuracy for each sub-class as calculated using threshold value *t *= 0.5.

**Class**	**Number of spots**	**Classification accuracy (%)**
not bad	35983	94.7
HIFI	942	98.7
LIFI	76	86.8
HIBI	987	96.5
LIBI	284	85.9
HIFI/HIBI	81	97.5
HIFI/LIBI	69	98.6
LIFI/HIBI	66	98.5
LIFI/LIBI	44	77.3
HIFI/LIFI	29	89.7
HIFI/LIFI/HIBI	62	100.0

The presented MASQOT approach was compared to three existing quality control methods: the composite quality score q_com _proposed by Wang *et al *[8], the mean-median correlation factor mm_corr _evaluated by Tran *et al *[9] and the coefficient of variation (CV) parameter CV_spot _evaluated by Sauer *et al *[10]. Threshold values for all quality control parameters were set to achieve maximum overall classification accuracy. The result, shown in table 3, demonstrates that the MASQOT approach provides a greater level of class discrimination for the POP2 test set compared to the remaining evaluated quality control methods.

**Table 3:** Comparison to other quality control methods. The presented quality control parameter CC_nb _was compared to the composite quality score q_com_, the mean-median correlation factor mm_corr _and the CV_spot _value. Threshold values for all quality control parameters were set to maximize overall classification accuracy. The classification accuracy was determined from classification of the POP2 test set.

**Quality control parameter**	**Threshold**	**Classification accuracy (%)**
CC_nb_	0.40	98.1%
q_com_	0.32	94.5%
mm_corr_	0.65	94.3%
CV_spot_	1.05	95.0%

## Discussion

cDNA microarray spot quality control is, in many aspects, a complex problem. Naturally, the automatic assessment of quality of each spot is highly reliant on the characterization of the spot. Incorrect approximations of the spatial location will affect the properties of the segmented foreground region, which in turn will influence the values of the quality control descriptors. In such a sequential process, where each step is dependent on the preceding steps, errors will propagate down-stream at a high rate. However, the most striking intricacy is perhaps the visual assessment, which is the foundation of this computer-based classification, where even experienced microarray users tend to disagree. As shown from the results presented here, it is the spots with unanimous visual quality assessment that are the most complicated to reproduce accurately. This disagreement stems from the more fundamental issue of defining 'quality' and in understanding the basal aspects that affect this quality.

The approach described here aims to assess the technical precision of each spot, which is believed to be linked to the biological accuracy (see [19] for a discussion regarding precision and accuracy in the microarray field). It should thus be stated, in this context, that lack of precision in a microarray spot measurement does not necessarily infer lack of accuracy. However, it is arguably reasonable to handle spots of questionable precision with specific care during the analysis procedure to aid the concluding biological interpretations.

The spot quality control assessment is commonly treated as a discrete problem (essentially, separating 'bad' spots from 'good' spots) but the spot quality varies on a continuous scale, ranging from very bad to very good. Instead of discarding spots, one might weight the spots according to the quality assessment. The concept of relative spot weights has previously been acknowledged in microarray normalization techniques (see, for instance [2,20]) but might also prove to be valuable in additional analysis steps. However, evaluation of the usage and validity of spot weights based on the quality assessments provided here remains the scope of a future paper.

The rate of accuracy in prediction of the true positives (bad spots predicted as bad) and the true negatives (not bad spots predicted as not bad) are illustrated separately since these accuracies are not consistently of equal importance. For instance, depending on the user and the question at hand, it might be more important to avoid the risk of removing spots of decent quality than to eliminate all of the bad spots from the data set. Simply using the overall classification accuracy could be rather illusive, merely since the number of bad spots is much lower than the number of not bad spots in a typical data set.

The methodology presented here is based on the scaled sum of the intensities from both channels but can, with minor adjustments, also be based on single channel intensities. By using the scaled channel intensity levels, one avoids the risk of drowning information, in particular when there is a great difference in intensity level between the channels. In addition, the presented approach greatly resembles the visual illustrations of the spots which, by design, will provide an advantage in finding correlation between the visual quality assessment and the spot descriptors. Furthermore, it is more feasible for the average user to achieve a per-spot quality measure than a per-channel quality measure since this avoids raising questions with regard to what to do when only one channel is of low or moderate quality.

The recent advances in spot quality control have clearly shown that a good explanation of training data is possible using several different methodologies of varying complexity. However, very few efforts have been made to evaluate further aspects of the quality (for instance, more refined descriptors) and, most importantly, the reproducibility of the classification on external data. External reproducibility has been the major aim here, partly overshadowing the aim of internal reproducibility on the training data, which is shown by the clear agreement in accuracy between the independent POP2 training set and the POP2 test set.

## Conclusion

The presented MASQOT technique provides a robust methodology for semi-automated cDNA microarray spot quality control with high accuracy of training data as well as external data compared to other evaluated methods. The MASQOT methodology generates non-discrete values of spot quality which can be utilized as weights in subsequent analysis procedures as well as to discard spots of undesired quality using the proposed threshold values.

## Methods

### Microarray preparation

Samples for the microarray slides used in this paper originate from an experiment of *Populus tremula *leaves, investigating regulation of leaf development (Sjödin *et al*, in preparation). The utilized microarray layout, referred to as POP2, consist of 25 278 single spotted cDNA clones from a recent assembly of more than 100 000 expressed sequence tags (ESTs) from the *Populus *genus [21]. All sequence information is available in the online sequence resource PopulusDB [22] and a full array layout is available for download from the online microarray resource UPSC-BASE [23].

Ten out of a total of 28 POP2 slides were randomly chosen for classification and were subsequently grouped into two equally large sets of five slides each; the POP2 training set and POP2 external test set. See additional file 2 for a complete list of the POP2 microarray slides used here. All POP2 slides were printed using a QArray arrayer (Genetix, Hampshire, U.K.). The preparation, labeling and hybridization of cDNA clones and mRNA samples were carried out according to the protocol described by Smith *et al *[24]. The arrays were scanned on a ScanArray 4000 (Perkin-Elmer Wellesley, MA) at 5 μm resolution to obtain raw image files for the red-fluorescent dye Cy5 and the green-fluorescent dye Cy3. All POP2 raw image files are available online for download at the UPSC-BASE microarray database [23] from experiment number 0013.

### Image analysis

The workflow from scanned cDNA images to computer-based classification was separated into seven sub-procedures, outlined below and illustrated in figure 1.

*1. Image merging *generated a combined image from the intensity measurements of both the red-fluorescent dye Cy5 and the green-fluorescent dye Cy3, which was used in the subsequent gridding and segmentation steps.

*2. Gridding *attempted to identify the precise spatial center of the spots on the scanned microarray images.

*3. Segmentation *classified the pixels as either representing the cDNA expression level (foreground pixels) or an estimation of the local noise level (background pixels). In addition, a thin strip of pixels in the boundary region between the two segments (border pixels) was identified.

*4. Information extraction *refers to the characterization of the foreground and background regions from the segmentation process. In general terms, information extraction should provide a description of each region that is relevant in some sense (for instance, the spatial location of the foreground region or the foreground intensity level.) The focus here was on features that captured the overall quality of the spot.

*5. Manual classification *provided a measure of the spot quality by means of visual inspection carried out by three experienced microarray users.

*6. Computer-based classification *of spot quality (the *training phase*) generated a model for the differences between the spot quality classes using discriminant analysis based on the PLS regression method (PLS-DA).

*7. Verification *of the computer-based classification (the *test phase*) validated the predictive ability of the model using processed data not included in the training phase.

### Image merging

Both segmentation and gridding were based on a combined eight-bit image constructed from the intensity measurements of the red-fluorescent dye Cy5 and the green-fluorescent dye Cy3. The merged eight-bit image lacks some details of the original images but is computationally efficient, in particular concerning memory requirements. Details of the utilized damping and scaling procedures are described by Yang *et al *[5] and briefly outlined below.

• The intensity levels in both images were square-root transformed. The square-root transform utilizes damping, which ensures that the relative impact of high-intensity pixels is decreased during gridding and segmentation.

• Median intensity values were computed from the transformed images.

• A joint intensity value was calculated using the sum of the square-root transformed intensities from both channels scaled according to the median values, respectively.

• Intensity values greater than 255 were truncated.

### Gridding

Approximate spatial centers of each spot, referred to as the *grid points*, were manually located using an in-house developed Java application. This procedure is the only step in the classification process that requires user intervention. A more precise midpoint of the foreground region was found using a square pixel mask with the expected spot diameter (100 μm for the POP2 data) surrounding the initial grid point. The pixel mask was spatially reallocated in all directions, deviating at most 30 μm from the initial grid point, and the center position of the square pixel mask containing the highest total sum of intensities was selected as seed point.

### Segmentation

The employed segmentation method was an implementation of the seeded region growing (SRG) algorithm, initially proposed by Adams and Bischof [18]. The SRG method has earlier been utilized in microarray spot segmentation by Yang *et al *[5]. Implementation details are available in additional file 3. The result from the segmentation process was a pixel mask categorizing each pixel into one of the four groups {foreground, border, background, un-assigned}. Each spot thus consisted of a distinct foreground region with the following characteristics:

• All pixels within the foreground region were spatially connected.

• No pixels overlapped with the foreground region of another spot.

• Minor fluctuations in intensity level within the region were accepted.

• The maximum Euclidean distance between any two pixels in the foreground region was restricted.

• Spot circularity was *not *assumed.

### Information extraction

The data utilized in the information extraction originates from the sum of the raw intensities of both the Cy5 and the Cy3 channels scaled according to the respective median intensity value. The scaling was applied to decrease the impact of the channel demonstrating the highest median intensity value. A large set of different features were extracted which were believed to be linked to spot quality and these were subsequently used in the upcoming computer-based classification. For purposes of repeatability and applicability to various types of microarray slides, all descriptors were corrected by an approximation of the slide background mean based on the mean intensity level of the local background regions from all spots on the slide.

Furthermore, spots where the saturation contents in at least one of the channels exceeded 10% of the total number of pixels, as suggested by Wang *et al *[8], were not included in the classification.

A complete table of all extracted descriptors including a description or definition is available in additional file 1. The descriptors aimed to capture foreground and background variability properties, spot morphology and foreground intensity and density properties.

### Manual classification

The spots from ten POP2 slides were independently inspected by three experienced microarray users and assigned to the two quality categories {bad, not bad}. The spots in the bad category consisted of all the spots that were classified as bad by at least one of the experienced users while the remaining spots were categorized as not bad.

During the visual classification, the experienced users worked according to four basic rules of thumb related to the technical precision of each spot.

• The signal within the foreground region should have low variability.

• The foreground region should be circular.

• The foreground region should be spatially located at the expected position.

• The background region should have low variability and low intensity level compared to the global slide background.

The relation between these, that is, how much each factor was allowed to deviate, alone and in combination with other factors, was the task for the multivariate classification model. The utilized data sets, subsequent to segmentation, are available at additional file 4 (training set) and additional file 5 (test set). A summary of the manual classifications as performed by the experienced users is available in additional file 6.

The POP2 slides were randomly partitioned into two equally large sets of five slides each; the POP2 training set and the POP2 test set. For classification and evaluation purposes, the bad spots of the training set were subsequently divided into different sub-classes based on visual properties as described in table 1. The HIFI and LIFI sub-classes were used as representatives of the pure foreground issues (FI) during classification training. Analogously, the HIBI and LIBI sub-classes were used as representatives of the pure background issues (BI) during classification training. Typical examples of the described sub-classes can be found in additional file 7.

### Computer-based classification

The computer-based classification was performed using PLS-DA as implemented in SIMCA-P+ 10.0 (Umetrics AB, Umeå, Sweden). Cross-validation [25] with seven groups was used to determine the number of latent variables. Prior to analysis, all descriptors were column-wise mean-centered and scaled to unit variance (UV) by dividing each descriptor with the standard deviation of the descriptor. The UV scaling procedure in combination with mean-centering translates the distribution of each descriptor to unit variance. Results and model statistics from the PLS-DA training phase are described in additional file 8.

Classification training was based on discriminant analysis of subsets of the foreground issues (FI) class, the background issues (BI) class and the not bad class. See table 1 for a more detailed description of the available sub-classes. Prior to discriminant analysis, a representative subset of each class consisting of 355 spots each was selected using D-optimal design [26] in order to eliminate the large differences in data set size between the three classes. See additional file 9 for the designed data set and additional file 10 for details regarding the D-optimal design.

Classification accuracies were calculated using equation 1, where *n *is the number of observations; *t *is a threshold value and *x *the predicted class conformity values for a given set of spots. Equation 1 utilizes the *corr*_*pred*_*(i, y, t) *function that returns 1 if *i *∈ {bad} and y < t *or *if *i *∈ {not bad} and y ≥ t; or 0 otherwise.

predacc(x,t)=100n∑i=1ncorrpred(i,xi,t)     (1)
 MathType@MTEF@5@5@+=feaafiart1ev1aaatCvAUfKttLearuWrP9MDH5MBPbIqV92AaeXatLxBI9gBaebbnrfifHhDYfgasaacH8akY=wiFfYdH8Gipec8Eeeu0xXdbba9frFj0=OqFfea0dXdd9vqai=hGuQ8kuc9pgc9s8qqaq=dirpe0xb9q8qiLsFr0=vr0=vr0dc8meaabaqaciGacaGaaeqabaqabeGadaaakeaacqWGWbaCcqWGYbGCcqWGLbqzcqWGKbazdaWgaaWcbaGaemyyaeMaem4yamMaem4yamgabeaakiabcIcaOiabdIha4jabcYcaSiabdsha0jabcMcaPiabg2da9maalaaabaGaeGymaeJaeGimaaJaeGimaadabaGaemOBa4gaamaaqahabaGaem4yamMaem4Ba8MaemOCaiNaemOCai3aaSbaaSqaaiabdchaWjabdkhaYjabdwgaLjabdsgaKbqabaGccqGGOaakcqWGPbqAcqGGSaalcqWG4baEdaWgaaWcbaGaemyAaKgabeaakiabcYcaSiabdsha0jabcMcaPaWcbaGaemyAaKMaeyypa0JaeGymaedabaGaemOBa4ganiabggHiLdGccaWLjaGaaCzcamaabmaabaGaeGymaedacaGLOaGaayzkaaaaaa@6051@

## List of abbreviations used

**PLS **Partial Least Squares

**PLS-DA **Partial Least Squares Discriminant Analysis

**SRG **Seeded Region Growing

**ROC **Receiver Operating Characteristics

**EST **Expressed Sequence Tag

## Authors' contributions

MB implemented the segmentation and gridding tools, performed visual classification, implemented the D-optimal design, conceived and generated the classification model and drafted the manuscript. DE generated the microarray slides, performed visual classification and helped to draft the manuscript. AS conceived the study, collected leaf samples, performed visual classification and helped to draft the manuscript. MS, SJ, HA and JT supervised the project. All authors read and approved the final manuscript.

## Supplementary Material

Additional File 1 Definition of the employed spot descriptors. Provides a definition of the employed spot descriptors used to assess the quality of each spot. Click here for file

Additional File 2 A list of the employed POP2 slides. Provides a list of the employed POP2 slides. Click here for file

Additional File 3 Implementation details of the segmentation process. Provides in-depth information regarding the implementation of the seeded region growing (SRG) algorithm. Click here for file

Additional File 4 The processed POP2 training data. Provides the processed POP2 training data set, which contains per-spot values of all descriptors employed here. Click here for file

Additional File 5 The processed POP2 test data. Provides the processed POP2 test data set, which contains per-spot values of all descriptors employed here. Click here for file

Additional File 6 Manual quality assessments. Provides a summary of the quality assessments as classified by the three experienced microarray users. Click here for file

Additional File 7 Visual representations of the sub-classes of bad spots. Provides images of typical examples of the 4 main sub-classes of bad spots. Click here for file

Additional File 8 Details of the PLS-DA model. Provides details and statistics from the utilized PLS-DA model. Click here for file

Additional File 9 The designed subset of the POP2 training data. Provides the processed and filtered POP2 training data set, containing only the spots from the not bad, FI and BI classes which were selected according to the D-optimal design. Click here for file

Additional File 10 Description of the utilized D-optimal design. Provides information regarding generation of the D-optimal design used in to select subsets of the three classes. Click here for file
