# Supplementary material for: MASQOT: a method for cDNA microarray spot quality control
Source: BMC Bioinformatics. 2005 Oct 13;6:250. doi: 10.1186/1471-2105-6-250 (PMC1276784; doi:10.1186/1471-2105-6-250)
Supplement: Additional File 1 — Definition of the employed spot descriptors. Provides a definition of the employed spot descriptors used to assess the quality of each spot. [file 1471-2105-6-250-S1.pdf]

Definition of descriptors used in the MASQOT methodology

Feature name	Feature description
FG |Ch1 - Ch2| CV	The CV value for the absolute pixel-wise difference between the channels.
FG % > |FG Mean +- 5SD|	The percent of foreground pixels outside the foreground intensity mean ± 5 standard deviations.
FG % > |FG Mean +- 6SD|	The percent of foreground pixels outside the foreground intensity mean ± 6 standard deviations.
FG % > |FG Mean +- 7SD|	The percent of foreground pixels outside the foreground intensity mean ± 7 standard deviations.
FG % > |FG Mean +- 8SD|	The percent of foreground pixels outside the foreground intensity mean ± 8 standard deviations.
FG % > |FG Mean +- 9SD|	The percent of foreground pixels outside the foreground intensity mean ± 9 standard deviations.
FG % > |FG Mean +- 10SD|	The percent of foreground pixels outside the foreground intensity mean ± 10 standard deviations.
FG % > BG Mean + 1 SD	The percent of foreground pixels greater than the local background mean plus one standard deviation (measured from the local background).
FG Circularity Approx.	An approximate estimate of circularity calculated as $100 \cdot \frac{\max(w,h)}{\min(w,h)}$ where w denotes the spot width and h denotes the spot height.
FG Circularity Loose	Circularity estimated using $\frac{100 \cdot A_o}{A_o + A_{no}}$ where A_o denotes the overlapping area and A_{no} denotes the non-overlapping area between the segmented foreground region and a perfect circle centered at the same nominal center with the same area.

Feature name	Feature description
`% DC vs NBorder`	Let E_d denote borders created due to a restriction in Euclidean distance towards the seed point. Let E_n denote the border pixels which are not complete engulfed by foreground pixels and/or other border pixels. The '`% DC vs NBorder`' variable is then defined as $\frac{100 \cdot E_d}{E_n}$
`% DC vs Total Border`	Let E_d denote borders created due to a restriction in Euclidean distance towards the seed point. Let E_t denote all the border pixels. The '`% DC vs Total Border`' variable is then defined as $\frac{100 \cdot E_d}{E_t}$
`FG SD / BG Mean GM`	The standard deviation of intensities within the foreground region corrected by the global background mean of the current slide.
`FG CV / FG CV GM`	The CV value of the intensities within the foreground region corrected by the global CV mean of the current slide.
`FG CV / BG Mean GM`	The CV value of the intensities within the foreground region corrected by the global background mean of the current slide.
`|FG Area - FG Area GM|`	The absolute difference in area (μm^2) between the current foreground area and the global area mean of the current slide.
`BG SD / BG Mean GM`	The standard deviation of intensities within the background region corrected by the global background mean of the current slide.
`BG CV / BG CV GM`	The CV value of the intensities within the background region corrected by the global CV mean of the current slide.

**Feature name**	**Feature description**
`BG CV / BG Mean GM`	The CV value of the intensities within the background region corrected by the global background mean of the current slide.
`|BG Area – BG Area GM|`	The absolute difference in area (μm^2) between the current background area and the global area mean of the current slide.
`FG Max / BG Mean GM`	The maximum intensity within the foreground region corrected by the global background mean of the current slide.
`BG Max / BG Mean GM`	The maximum intensity within the background region corrected by the global background mean of the current slide.
`FG Mean / BG Mean GM`	The mean intensity of the foreground region corrected by the global background mean of the current slide.
`BG Mean / BG Mean GM`	The mean intensity of the background region corrected by the global background mean of the current slide.
`FG Quantile 95% / BG Mean GM`	The 95% quantile intensity within the foreground region corrected by the global background mean of the current slide.
`FG Quantile 99% / BG Mean GM`	The 99% quantile intensity within the foreground region corrected by the global background mean of the current slide.
`BG Quantile 95% / BG Mean GM`	The 95% quantile intensity within the background region corrected by the global background mean of the current slide.
`BG Quantile 99% / BG Mean GM`	The 99% quantile intensity within the background region corrected by the global background mean of the current slide.

Feature name	Feature description
`FG Quadrant Diff. Mean / BG Mean GM`	Let Δ_f denote the intensity difference between the foreground quadrant containing the maximum intensity mean and the foreground quadrant containing the minimum intensity mean. Let $\bar{g}_b$ denote the global background mean of the current slide. The '`BG Quadrant Diff. Mean / BG Mean GM`' variable is then defined as $\frac{\Delta_f}{\bar{g}_b}$
`BG Quadrant Diff. Mean / BG Mean GM`	Let Δ_b denote the intensity difference between the background quadrant containing the maximum intensity mean and the background quadrant containing the minimum intensity mean. Let $\bar{g}_b$ denote the global background mean of the current slide. The '`BG Quadrant Diff. Mean / BG Mean GM`' variable is then defined as $\frac{\Delta_b}{\bar{g}_b}$
`FG Uniformity / FG Uniformity GM`	Let f_{max} denote the maximum intensity within the foreground region. Let f_{min} denote the minimum intensity within the foreground region. Denote the uniformity u as $u = (f_{max} - f_{min})/(f_{max} + f_{min})$. The '`FG Uniformity / FG Uniformity GM`' variable is defined as the uniformity of the current foreground region corrected by the global mean within the slide.
`FG Roundness / FG Roundness GM`	Let A denote the area of the current foreground region and P the perimeter. Let the roundness r of a spot be defined as $r = 100(4\pi A)/P^2$. The '`FG Roundness / FG Roundness GM`' is defined as the roundness of the current foreground region corrected by the global mean within the slide.
