# Supplementary material for: MASQOT: a method for cDNA microarray spot quality control
Source: BMC Bioinformatics. 2005 Oct 13;6:250. doi: 10.1186/1471-2105-6-250 (PMC1276784; doi:10.1186/1471-2105-6-250)
Supplement: Additional File 3 — Implementation details of the segmentation process. Provides in-depth information regarding the implementation of the seeded region growing (SRG) algorithm. [file 1471-2105-6-250-S3.pdf]

Segmentation using the Seeded Region Growing (SRG) algorithm

The segmentation process was performed on the basis of print-tip groups. Applied sequentially on each print-tip group, the segmentation started with a fixed number of foreground seeds centered at the seed point generated from the gridding procedure. A foreground seed was here defined as a square 30 μm by 30 μm region forming the initial foreground region for each spot. Foreground regions were subsequently extended using the procedure outlined below.

- Pixels not assigned as `foreground` pixels but neighboring to at least one `foreground` pixel were considered putative `foreground` pixels. Putative `foreground` pixels were sequentially added to the proximal foreground region in descending order based on the respective rank. The rank of each putative pixel was set inversely proportional to the absolute intensity difference to the intensity mean of the proximal foreground region. This is analogous to extending the foreground region with the neighboring pixel containing the intensity level closest to the mean intensity level of the region.
- Neighboring pixels were rejected as putative `foreground` pixels unless both of the following criteria were met:
    - Let the distance between two intensity levels I_1 and I_2 be denoted as $|I_1 – I_2|$. The distance between the neighboring pixel and the intensity mean of the proximal foreground region must be shorter than the distance between the neighboring pixel and the estimated print-tip background. Background of the current print-tip group was estimated using the mean intensity value of the fraction of pixels within the print-tip region which were believed to be non-foreground according to *a priori* information regarding the estimated size of the spots as well as the spatial distance between the spots. For the slides processed here, the background estimation was defined as the mean of the pixel intensities in the print-tip group that were below or equal to the print-tip group median intensity.

- The neighboring pixel was spatially located inside a fixed Euclidean distance from the seed point, set to 90 µm for the `POP2` data sets.

The segmentation process terminated when no more putative `foreground` pixels existed. An illustration of an intermediate step in this procedure is demonstrated in figure S1.

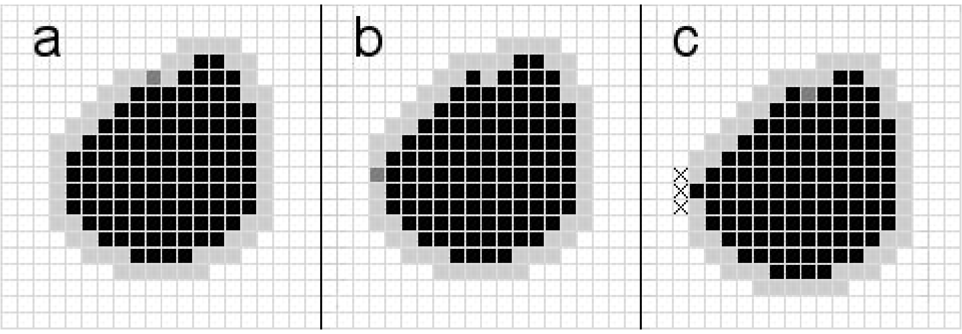

Figure S1. Illustration of intermediate pixel assignments during the segmentation process. Black squares depict `foreground` pixels, light grey squares depict putative `foreground` pixels, crossed-out squares depict pixels that are not accepted as putative foreground pixels and white squares depict `un-assigned` pixels. The dark grey square depicts the pixel with the best rank which will subsequently be encompassed in the foreground region. In the first subsection **a** (left), the highest ranked pixel has been identified and will be added to the foreground region as illustrated in subsection **b** (center). In addition, the set of putative `foreground` pixels in **b** are expanded to include all neighboring pixels formerly `un-assigned`. In subsection **c** (right) a `foreground` pixel is added but the neighboring pixels are not accepted as putative `foreground` pixels due to a higher rank towards the global background estimate compared to the current foreground intensity mean. The process terminates when all neighboring pixels are determined to be non-`foreground`.

The result from the segmentation process was a pixel mask categorizing each pixel into one of the four groups `{foreground, border, background, un-assigned}`. Each spot thus consisted of a distinct foreground region with the following characteristics:

- All pixels within the foreground region were spatially connected.
- No pixels overlapped with the foreground region of another spot.
- Minor fluctuations in intensity level within the region were accepted.
- The maximum Euclidean distance between any two pixels in the foreground region was restricted.
- Spot circularity was *not* assumed.

The border region was defined as a layer of pixels of width 10 μm immediately outside the foreground region. In this way, the region formed a coating between the foreground region and the background region used to estimate potential intensity fluctuations in the foreground/background boundary. The local background region was a layer of pixels of width 30 μm immediately outside the border region used to estimate the local noise level. The local background region might overlap with the local background of other spots but might not include `foreground` pixels assigned to other spots. Un-assigned pixel regions contained the pixels not categorized as `foreground`, `border` or `background` and were not used in any subsequent analysis. A graphical representation of a hypothetical segmented spot can be found in figure S2.

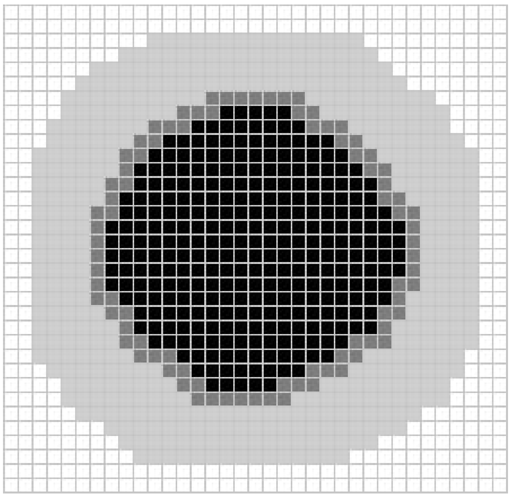

Figure S2. Visual representation of a segmented spot. Pixels are of the type `foreground` pixels (black squares), `border` pixels (dark grey squares), `background` pixels (light grey squares) and `un-assigned` pixels (white squares). The depicted border region is a one-pixel wide layer outside the foreground region while the background region is a four-pixel wide layer surrounding the border region.

Unexpressed control spots on the array were utilized to determine the global noise level of the processed slides. For the `POP2` layout, there are a total of 809 such spots dispersed across each array. The intensity of each of the control spots was estimated using the median intensity of an approximate spot region as determined from the gridding phase. The global signal intensity threshold for each slide was set at the 95% quantile of the channel-wise summed intensities of the unexpressed control spots. The results are shown in table S1. All spots where the estimated noise level exceeded the

channel-wise summed median foreground intensity were characterized as noise and not included in further analyses.

Table S1. Slide noise levels as estimated using unexpressed control spots. The signal threshold is based on the channel-wise summed intensity levels.

**Slide identifier**	**Noise threshold**
12623358	705
12623072	462
12623431	323
12623496	770
12618706	236
12618709	303
12623074	448
12623364	684
12623488	451
12623498	417
