# Supplementary material for: MASQOT: a method for cDNA microarray spot quality control
Source: BMC Bioinformatics. 2005 Oct 13;6:250. doi: 10.1186/1471-2105-6-250 (PMC1276784; doi:10.1186/1471-2105-6-250)
Supplement: Additional File 7 — Visual representations of the sub-classes of bad spots. Provides images of typical examples of the 4 main sub-classes of bad spots. [file 1471-2105-6-250-S7.pdf]

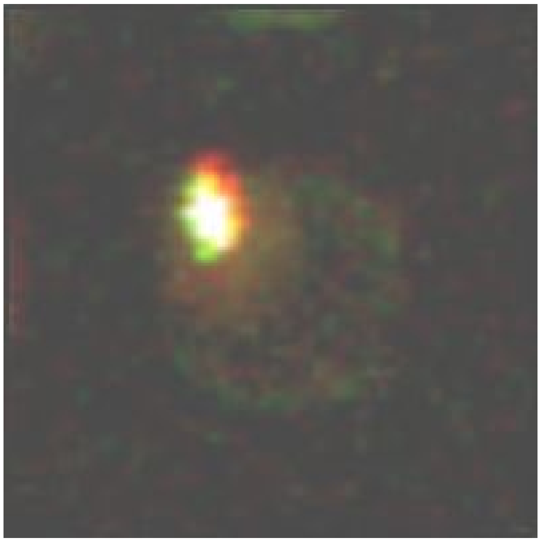

High-Intensity Foreground Issue (`HIFI`)

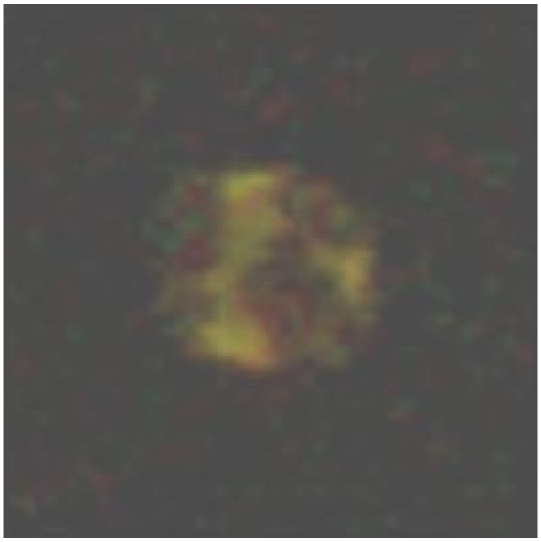

Low-Intensity Foreground Issue (`LIFI`)

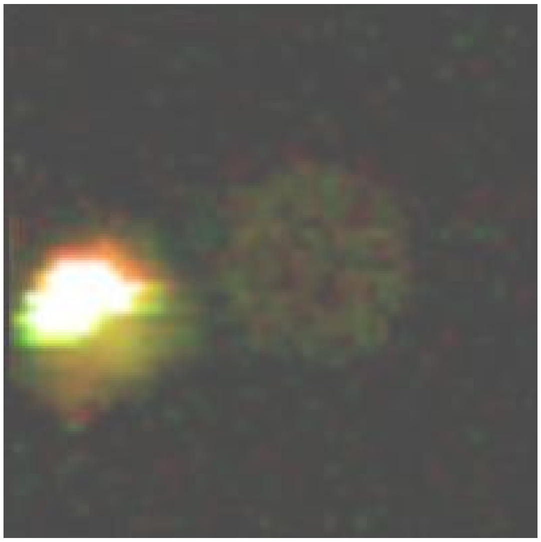

High-Intensity Background Issue (`HIBI`)

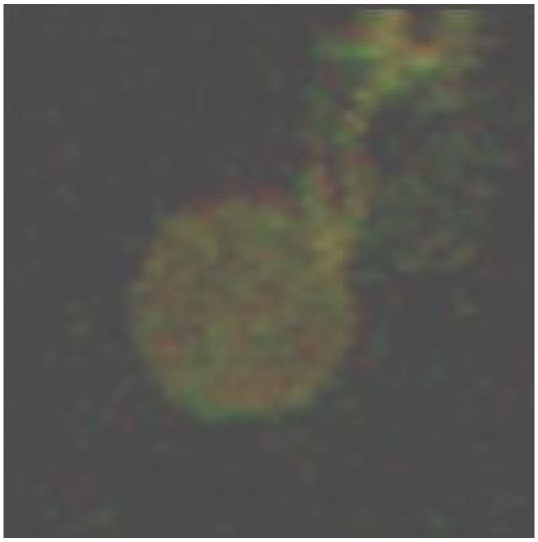

Low-Intensity Background Issue (`LIBI`)
