# Supplementary material for: MASQOT: a method for cDNA microarray spot quality control
Source: BMC Bioinformatics. 2005 Oct 13;6:250. doi: 10.1186/1471-2105-6-250 (PMC1276784; doi:10.1186/1471-2105-6-250)
Supplement: Additional File 8 — Details of the PLS-DA model. Provides details and statistics from the utilized PLS-DA model. [file 1471-2105-6-250-S8.pdf]

Brief overview of PLS and PLS-DA

PLS and PLS-DA are methods for relating two data matrices **X** and **Y** using multivariate linear regression estimated by means of partial least squares. A set of latent variables are calculated which aim to maximize the covariance between the **X** and **Y** matrices. The data matrix **X** consists, in this case, of the spot descriptors described in the file `Descriptor_definitions.pdf`. The data matrix **Y** in PLS-DA is a matrix defining class belonging, which is set to either 1 (belongs to class) or 0 (does not belong to class).

Definitions

For simplicity, both **X** and **Y** are column centered in the equations below.

- R_X^2 is a measure of explained variances in the **X** block after model fitting, calculated using the residuals of the **X**-block:

$$R_X^2 = 1 - \frac{\sum_{i=1}^{K}\sum_{i=1}^{N}\left(X_{observed} - X_{calculated}\right)^2}{\sum_{i=1}^{K}\sum_{i=1}^{N}X_{observed}{}^2} \qquad (1)$$

  The value of R_X^2 ranges from 0 (no variance explained) to 1 (all variance explained).

- R_Y^2 is the corresponding value for the **Y** (class) block.

$$R_Y^2 = 1 - \frac{\sum_{i=1}^{M}\sum_{i=1}^{N}\left(Y_{observed} - Y_{calculated}\right)^2}{\sum_{i=1}^{M}\sum_{i=1}^{N}Y_{observed}{}^2} \qquad (2)$$

- Q_Y^2 is a measure of the predicted variance of a model, calculated using cross-validation. During cross-validation, a subset of data is excluded, a model is fitted to the remaining data and subsequently the excluded data is predicted from the (reduced) model. The process is repeated component-wise for each subset and yields an estimate of the predictive ability of a model. The main difference between R_Y^2 and Q_Y^2 is thus that the numerator in equation 2 and 3 is part of the model for R_Y^2 (equation 2) but not for Q_Y^2 (equation 3).

$$Q_Y^2 = 1 - \frac{\sum_{i=1}^{M}\sum_{i=1}^{N}\left(Y_{observed} - Y_{calculated}\right)^2}{\sum_{i=1}^{M}\sum_{i=1}^{N}Y_{observed}{}^2} \qquad (3)$$

PLS-DA model information

The three-class PLS-DA training phase resulted in a model with 7 latent variables (by cross-validation) with R_X^2 =0.715, R_Y^2 =0.646 and Q^2 = 0.609.

Coefficients

The coefficient matrix **B** from the PLS model is used to predict a value of an unknown observation according to the formula **Y** = **XB**. The coefficient matrix when **X** is mean-centered and scaled to unit-variance is available in table S1.

Table S1. Regression coefficients for the `not bad`, `FI` and `BI` classes, respectively. Coefficients are estimated from the PLS-DA model when the descriptor matrix **X** is centered and scaled to unit variance.

	not bad	FI	BI
FG |Ch1 - Ch2| CV	0.074570	-0.052037	-0.021817
FG % > |FG Mean +- 5SD|	-0.191724	0.246505	-0.057539
FG % > |FG Mean +- 6SD|	-0.124827	0.189432	-0.066637
FG % > |FG Mean +- 7SD|	-0.099143	0.145736	-0.048169
FG % > |FG Mean +- 8SD|	-0.119419	0.164249	-0.046635
FG % > |FG Mean +- 9SD|	-0.093627	0.118394	-0.026098
FG % > |FG Mean +- 10SD|	-0.165667	0.167000	-0.003342
FG % > BG Mean + 1SD	-0.165498	0.062282	0.102061
FG Circularity Approx.	-0.012545	0.003824	0.008641
FG Circularity Loose	0.161752	-0.038488	-0.122318
% DC vs NBorder	-0.068896	-0.000241	0.068869
% DC vs Total Border	0.006702	0.004088	-0.010798
FG SD / BG Mean GM	-0.004454	-0.005413	0.009893
FG CV / FG CV GM	0.011465	-0.031000	0.019832
FG CV / BG Mean GM	0.166647	-0.087783	-0.077497
|FG Area - FG Area GM|	0.032375	0.019176	-0.051581
BG SD / BG Mean GM	0.035878	-0.000579	-0.035155
BG CV / BG CV GM	-0.432117	-0.017587	0.448157
BG CV / BG Mean GM	0.038237	-0.054427	0.016783
|BG Area - BG Area GM|	-0.179874	0.027153	0.151797
FG Max / BG Mean GM	-0.013686	-0.022821	0.036640
BG Max / BG Mean GM	-0.082495	0.000097	0.082074
FG Mean / BG Mean GM	0.142658	0.014783	-0.157004
BG Mean / BG Mean GM	-0.209449	0.001522	0.207095
FG Quantile 95% / BG Mean GM	-0.077339	0.121958	-0.045915
FG Quantile 99% / BG Mean GM	0.031747	-0.033242	0.001890
BG Quantile 95% / BG Mean GM	-0.140898	-0.007654	0.148063
BG Quantile 99% / BG Mean GM	0.013523	-0.013206	-0.000157
FG Quadrant Diff. Mean / BG Mean GM	-0.120245	0.074368	0.044801
BG Quadrant Diff. Mean / BG Mean GM	-0.045617	-0.015425	0.060989
FG Uniformity / FG Uniformity GM	-0.039119	0.104925	-0.066815
FG Roundness / FG Roundness GM	-0.060835	-0.030975	0.091824
[Constant]	0.695535	0.721257	0.703540
