# Supplementary material for: MASQOT: a method for cDNA microarray spot quality control
Source: BMC Bioinformatics. 2005 Oct 13;6:250. doi: 10.1186/1471-2105-6-250 (PMC1276784; doi:10.1186/1471-2105-6-250)
Supplement: Additional File 10 — Description of the utilized D-optimal design. Provides information regarding generation of the D-optimal design used in to select subsets of the three classes. [file 1471-2105-6-250-S10.pdf]

Overview of D-optimal design

D-optimal design is a computer-generated design to select a subset from an existing set of observations according to a specific criterion. A typical case would be for a matrix A containing *K* observations describing all the information of a given experiment that, for various reasons, needs to be reduced to *N* observations. D-optimal design selects a matrix $X \subset A$ that maximizes the determinant of the X^TX matrix, where X consist of *N* observations from the A matrix. Thus, out of all possible ways of selecting *N* out of *K* observations, forming the matrix X, the combination with the highest numerical value of the determinant of the variance-covariance matrix X^TX will be selected. Maximizing the X^TX matrix is beneficial for the precision of the regression coefficients when employing least squares.

Implementation details

D-optimal design was implemented in the statistical programming environment `R`. A fixed number of observations (spots) was chosen prior to the design, selected as $N = 350$ observations + 5 center points = 355 observations for each class. The determinant of the X^TX matrix, where X contains $N = 350$ randomly selected rows, was calculated independently for the `not bad` and the considerably smaller `FI` and `BI` classes. The process was repeated 1 000 000 times for the `not bad` class and 10 000 times for the `FI` and `BI` class, respectively, to find a (local) maximum value of the determinant. The 5 center points were added subsequently to the D-optimal design.
